# Molecular diagnosis of acute and chronic infection of *Trypanosoma evansi* in experimental male and female mice

**DOI:** 10.4102/ojvr.v86i1.1638

**Published:** 2019-08-26

**Authors:** Tahani S. Behour, Shawky M. Aboelhadid, Wahid M. Mousa, Adel S. Amin, Saeed A. El-Ashram

**Affiliations:** 1 Biotechnology Research Unit, Animal Reproduction Research Institute, Giza, Egypt; 2 Department of Parasitology, Faculty of Veterinary Medicine, Beni Suef University, Beni Suef, Egypt; 3 College of Life Science and Engineering, Foshan University, Foshan, Guangdong Province, China; 4 Faculty of Science, Kafrelsheikh University, Kafr Elsheikh, Egypt

**Keywords:** *Trypanosoma evansi*, acute, chronic, mice, PCR, polymerase chain reaction, organs

## Abstract

*Trypanosoma evansi* is enzootic in camels in Egypt, and water buffaloes act as a reservoir for camel infection. Molecular techniques have contributed towards understanding the epidemiology of *T. evansi. Trypanosoma evansi* was detected in acute and chronic stages of the disease in male and female mice by polymerase chain reaction (PCR) using two primers. Two experiments were conducted. In experiment I, two groups consisting of 26 female and 26 male mice received 10^4^ trypanosome by I/P inoculation for each mouse. In experiment II, 42 female and 42 male mice were inoculated I/P with 10^2^ trypanosome/mouse. In addition, five mice were kept as uninfected control for each group. Mice were monitored daily for parasitaemia level during the pre-patent period using the micro-haematocrit centrifugation technique (MHCT) and conventional PCR. The primer pairs, (*Trypanosoma brucei*) TBR1/2 and TeRoTat1.2 (*T. evansi* Rode Trypanozoon antigen type [RoTat] 1.2), detected the infection after 24 hours earlier than MHCT in both experiments. The course of infection that was detected by MHCT revealed three waves of parasitaemia in female mice and two waves in male mice in the chronic stage of infection. In addition, PCR was able to detect *T. evansi* in different organs in the chronic stage (i.e. disappearance of parasite from blood). Application of the two primer sets on blood samples from camels showed that all samples were positive by TBR1/2 primers and only 32 of 44 were positive by TeRoTat1.2 primers. Acutely and chronically *Trypanosoma*-infected mice were detected by PCR in blood and organs. TBR1/2 primers were more sensitive than TeRoTat1.2 primers in detecting *Trypanosoma*-infected mice, and more reliable in detecting field-infected camels and excluding carrier animals.

## Introduction

*Trypanosoma evansi* belongs to the genus *Trypanosoma* and subgenus *Trypanozoon*, and is the first pathogenic trypanosome to be discovered globally. *Trypanosoma evansi* was hypothesised to originate from *Trypanosoma brucei* (TBR), by lacking genes necessary for mitochondrial development with loss of ability to undergo growth and differentiation in the insect vector and adaptation to a non-cyclic mode of transmission for the parasite (Carnes et al. 2015; Luckins 1988). *Trypanosoma evansi* has been able to spread rapidly by non-specific mechanical vectors, such as tabanids and stomoxes (Herrera et al. 2004; Otto et al. 2010). *Trypanosoma evansi* is enzootic in camels in Egypt, and water buffaloes act as a reservoir for camel infection (Elhaig, Youssef & El-Gayar 2013; Hilali et al. 2004). Surra is an insect-borne parasitic disease caused by *T. evansi* with wide distribution globally. *Trypanosoma evansi* infects a wide diversity of mammalian hosts, including animals and humans. However, camels, horses and dogs remain the most critical hosts for this parasite, and bovine hosts are very efficient reservoirs (Desquesnes et al. 2013; Fernandez et al. 2009; Rjeibi et al. 2015). The syndromes associated with *T. evansi* infection are severe and fatal, especially in the late stage of the disease. The disease varies from chronic to lethal acute accompanied with progressive weakness, emaciation, depletion, recurrent fever, enlarged lymph nodes and death (Omer, Mousa & Al-Wabel 2007; Saleh, Bassam & Sanousi 2009). The underestimation of the medical and economic impacts of *T. evansi* has contributed to its ability to spread silently via healthy carriers. Although *T. evansi* is inapparent in most instances, the parasite affects livestock productivity causing mortality, reduced animal production and reproduction performance, low carcass quality, and decreased animal strength (Desquesnes et al. 2013; Reid 2002). Diagnosis of *T. evansi* infection relies on the detection of the parasite in the blood or tissue fluids of infected animals. Parasitological techniques cannot always detect ongoing infections as the level of parasitaemia is often low and fluctuating, particularly during the chronic stage of the disease, which exhibits very low parasitaemia (Nantulya 1990). Consequently, the sensitivity and specificity of parasitological diagnostic tests are unacceptable in situations where confirmation of presence or absence of *T. evansi* in livestock is necessary prior to the introduction of new animals or after implementation of control and eradication programmes (Viljoen & Luckins 2012).

The nucleic acid-based assays are considered to be the most powerful tools for the detection of *T. evansi* in several animals and vectors (Sukhumsirichart et al. 2000). Molecular techniques enable researchers to identify and characterise the newly introduced *T. evansi* strains, detect mixed infections, study the disease epidemiology and understand the interaction between vectors and reservoirs (Fernandez et al. 2009). Therefore, the objective of this study was to compare and evaluate the sensitivity and specificity of the two primer sets in the detection of acutely and chronically *Trypanosoma*-infected male and female mice.

## Materials and methods

All procedures for animal infection, euthanasia and sample collections were approved by the Animal Reproduction Research Institute, Agricultural Research Center, Egyptian Committee.

### *Trypanosoma evansi* strain

*Trypanosoma evansi* strain (isolated from naturally infected camels) was obtained from the Department of Parasitology, Faculty of Veterinary Medicine, Cairo University. The parasite was propagated in laboratory mice and preserved in liquid nitrogen, according to Shumei et al. (1996), for further processing.

### Mice

Swiss albino mice (73 females and 73 males) weighing 25 g – 30 g were purchased from the Laboratory Animal Building, Research Institute of Ophthalmology, Giza. The mice were housed in a pathogen-free environment for 2 weeks prior to the initiation of experiments. The mice were subjected to feed and water *ad libitum*.

#### Experiment I

Two groups of mice, 26 females and 26 males, were kept separately. Twenty-one mice from each group received 10^4^ trypanosome/mouse by I/P inoculation (infective dose [ID]), according to Sharma et al. (2012). The other five mice from each group were served as uninfected control groups. Three mice were sacrificed daily from each group after infection. For each mouse, blood samples were collected from a tail vein to detect the pre-patent period and estimate the peak of parasitaemia and molecular assays. In addition, liver, spleen, kidneys, brain and ovaries or testis were collected on 1, 3, 5, and 7 days post-infections (DPI). A section from each organ was preserved at -20 °C for molecular examination.

#### Experiment II

Two groups of mice, 47 females and 47 males, were kept separately. Forty-two mice from each group were inoculated I/P with 10^2^ trypanosome/mouse (ID), according to Sharma et al. (2012). Five mice from each group were served as an uninfected normal control group. Three mice from each group were sacrificed from 1–7 DPI and were examined by micro-haematocrit centrifugation technique (MHCT) to detect the pre-patent period and estimate the peak of parasitaemia. All remaining mice were subsequently followed up biweekly for the assessment of parasitaemic waves during the course of infection by MHCT from peripheral blood. Samples were collected, as mentioned before (see experiment I), from each mouse during parasitaemic and aparasitaemic waves.

### Parasitological examination

All blood samples were collected from caudal vein blood in heparinised tubes and then centrifuged for 5 minutes at 5000 revolutions per minute. The capillary tubes were examined under the microscope (×10) for the detection of trypanosomes using MHCT (Woo 1970).

### Molecular detection of *Trypanosoma evansi* in blood and tissue samples of experimentally infected mice in both experiments

#### Deoxyribonucleic acid extraction from blood and tissue samples

DNA was extracted from each blood sample according to the procedure used by Sarataphan et al. (2007). Briefly, 50 *µ*L of each blood sample was lysed twice in 500 *µ*L of 0.1 M ammonium chloride and then centrifuged. The sediment was re-suspended in 50 *µ*L of 0.002% sodium dodecyle sulphate and 50 *µ*L of 5% Chelex-100^®^ (Sigma) suspension in TE buffer (10 mM Tris–HCl, 0.1 mM EDTA, pH 8.0).

The mixture was heated at 70 °C for 8 min followed by heating to 100 °C for 10 min. Finally, the mixture was centrifuged at 10 000 rpm for 5 min at room temperature, and the supernatant was stored at -80 °C for molecular detection of trypanosomes by polymerase chain reaction (PCR). For tissue samples, 0.5 g of each tissue was washed in 500 *µ*L of phosphate-buffered saline and then grinded in liquid nitrogen. The obtained pellet was suspended in 300 *µ*L of 10% Chelex-100^®^ suspension in TE buffer. The tissue samples were subsequently processed as stated above.

#### Conventional polymerase chain reaction sensitivity for detection of *Trypanosoma evansi*

To determine the detection limit of the standard PCR assay and establish a standard dilution for detection, 10-fold serial dilutions of known aliquots of *T. evansi* (1.0 × 10^6^ trypanosomes) were used for seeding the non-infected camel blood. Dilutions were subjected to DNA extraction as described above and then processed by conventional PCR as described below using TBR F-TBR R (Masiga et al. 1992) and (*T. evansi* Rode Trypanozoon antigen type) TeRoTat920 F-TeRoTat1070 R (Konnai et al. 2009) primer sets.

#### Deoxyribonucleic acid amplification by conventional polymerase chain reaction assays on blood and tissue samples from *Trypanosoma*-infected mice in both experiments

PCR analysis was done by using primer pairs TBR F-TBR R (Masiga et al. 1992) and TeRoTat920 F-TeRoTat1070 R (Konnai et al. 2009) in standard PCR procedures. Briefly, the PCR mixture of 25 *µ*L contained 2X Taq Master Mix, 25 pmol of each primer, 2 *µ*L of Deoxyribonucleic acid (DNA) template and up to 25 µL nuclease-free water were added. PCR using TBR F (5′-GAA TAT TAA ACA ATG CGC AG-3′) and TBR R (5′-CCA TTT ATT AGC TTT GTT GC-3′) was performed in a thermocycler (Nexus Gradient Eppendorf, Germany) as follows: an initial denaturation step at 95 °C for 4 min, 30 cycles of denaturation at 95 °C for 45 seconds, annealing at 52 °C for 45 s, and extension at 72 °C for 60 s and final extension at 72 °C for 10 min. PCR using TeRoTat920 F (5′-CTG AAG AGG TTG GAA ATG GAG AAG-3′) and TeRoTat1070 R (5′-GTT TCG GTG GTT CTG TTG TTG TTA-3′) was carried out in a thermocycler (Eppendorf Thermal Cycler, Germany) as follows: an initial denaturation step at 95 °C for 4 min, 35 cycles of denaturation at 95 °C for 60 s, annealing at 52 °C for 60 s, and extension at 72 °C for 60 s and final extension at 72 °C for 10 min. Amplicons were resolved on a 1.5% agarose gel stained with ethidium bromide (Sigma) and photographed under ultra violet (UV) light (Sambrook and Russell 2001). Positive control (*T. evansi* DNA) and negative control (reaction mixtures without DNA) were included in each PCR run.

#### Application of polymerase chain reaction assay on field blood samples from camels

Forty-four camels’ blood samples were collected in sterile heparinised tubes from different locations in Giza and Nobariya provinces for detection and isolation of *T. evansi.* Each sample was tested by MHCT, and 0.5 mL of blood was inoculated I/P into a laboratory mouse to investigate parasitic infection. DNA was extracted, and the PCR assay was conducted as mentioned before.

### Ethical considerations

Ethical approval was provided by the Committee at the Animal Reproduction Research Institute Agricultural Research Center, Egypt (23-2016).

## Results

### Following-up the course of infection in experimentally infected mice

**FIGURE 1:**
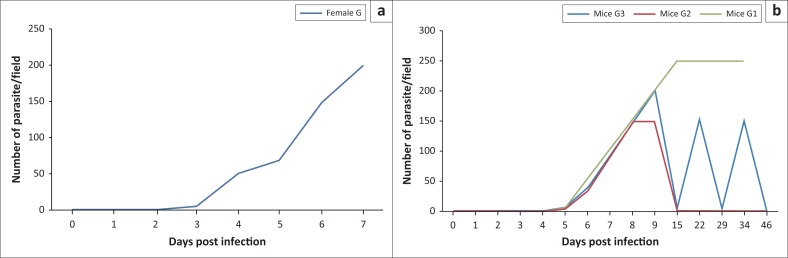
Infection in experimentally infected mice. The course of the parasitemia during the period of the experiment I (a) (10^4^ infective dose) and experiment II (b) (10^2^ infective dose) by micro-haematocrit centrifugation technique. The days post infection is plotted against the approximate no. of parasite in 50 *u*L blood. The plotted curves represent the different disease patterns obtained in mice during both experiments.

Different parasitaemic patterns were appeared in the infected groups according to the ID in mice. In experiment I, the pre-patent period (appearance of parasite in blood) was detected at 3 DPI and reached the peak of parasitaemia at 7 DPI (Figure 1, experiment I). In experiment II (Figure 1, experiment II), the pre-patent period was determined at 5 DPI and parasitaemia peaked at 9 DPI, and three infection patterns were observed as follows: six mice showed persistent parasitaemia and died within 12–34 DPI (G1). In contrast, other six mice showed the disappearance of parasitaemia at 15 DPI and remained stable at this level until the end of the experiment (G2). Twelve mice of the group (G3) displayed three waves of parasitaemia at 9, 22 and 34 DPI, with the disappearance of parasitaemia at 15, 29 and 46 DPI (Table 1).

**TABLE 1:** Summary of experimental infection.

Item	Experiment I	Experiment II
Infective dose	10^4^ Parasite/mouse	10^2^ Parasite/mouse
Survival duration of infected mice	7–9 days	12–22 days for male mice
		12–46 days for female mice
Parasitaemic waves	1	3
Chronic waves	None	3
Pre-patent period		
MHCT	3 days	5 days
PCR	24 h	24 h

MHCT, micro-haematocrit centrifugation technique; PCR, polymerase chain reaction.

### Detection limit of *Trypanosoma evansi* using conventional polymerase chain reaction

**FIGURE 2:**
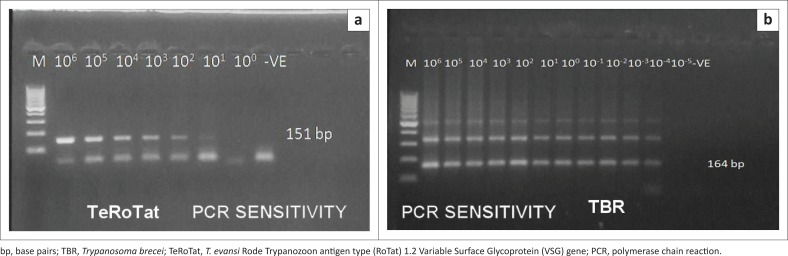
Polymerase chain reaction detection limit by conventional polymerase chain reaction. Lane M: 100 base pairs molecular weight Deoxyribonucleic acid marker. Lanes 1–12: different dilutions and -ve: negative control. *Trypanosoma brucei* 1/2 detects *Trypanosoma evansi* at lower dilutions (10^-4^ parasite/mL) than T. evansi Rode Trypanozoon antigen type 1/2 (10 parasite/mL of blood). (a) TeRoTat; (b) TBR.

PCR sensitivity was estimated using two primer sets TeRoTat1.2 and TBR1/2 with 10-fold serial dilution of 10^6^ parasites/mL. Using TeRoTat1.2 primer set, the minimal detection limit was 10 parasites/mL blood. No extra bands were amplified except the 151 bp (base pairs) target DNA band (Figure 2a). Employing TBR1/2 primer set, the lowest detection limit was 0.0001 parasites/mL blood despite the appearance of two extra non-specific bands in addition to the 164 bp, specifically amplified band (Figure 2b).

### Conventional polymerase chain reaction detection of *Trypanosoma evansi* in blood in both experiments

**FIGURE 3:**
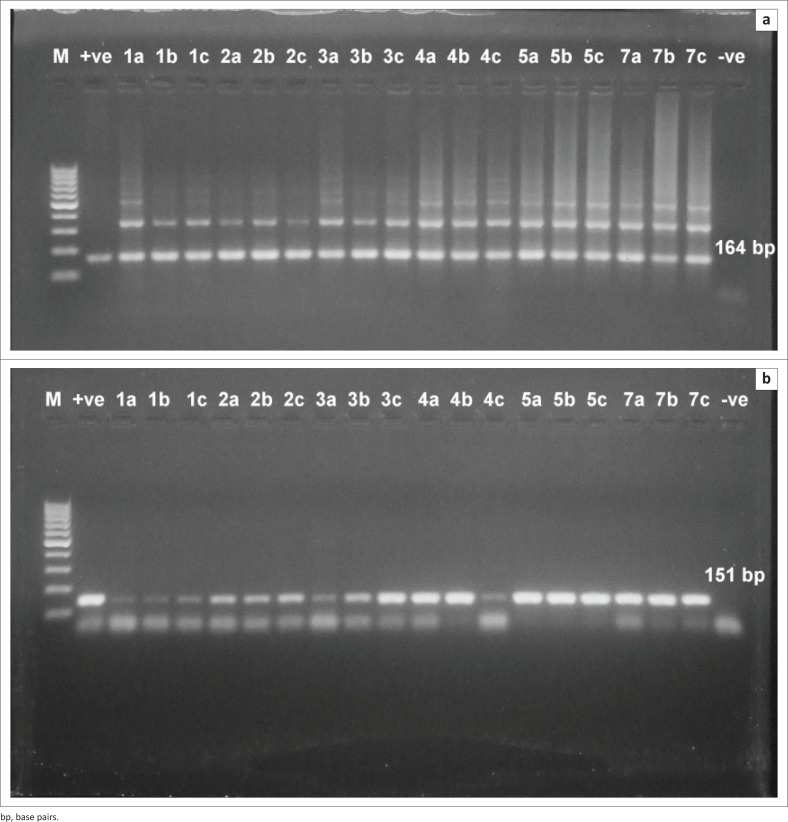
Direct polymerase chain reaction from mouse blood injected by 10^4^ trypanosome. (a) Polymerase chain reaction amplification using *Trypanosoma brucei* 1/2 primer sets. The band 164 base pairs was high intensity and consistently visible throughout the course of infection. (b) Polymerase chain reaction amplification using *T. evansi Rode Trypanozoon antigen type* 1.2 primer sets. The bands intensity was clearly observed and increased gradually from the first day post infection until the peak of parasitemia. M: 100 base pair molecular weight Deoxyribonucleic acid marker, +ve: positive control, -ve: negative control, Numbers: days post infection. Letters: mice were sacrificed at day 1, 3, 5 and 7 post infection.

**FIGURE 4:**
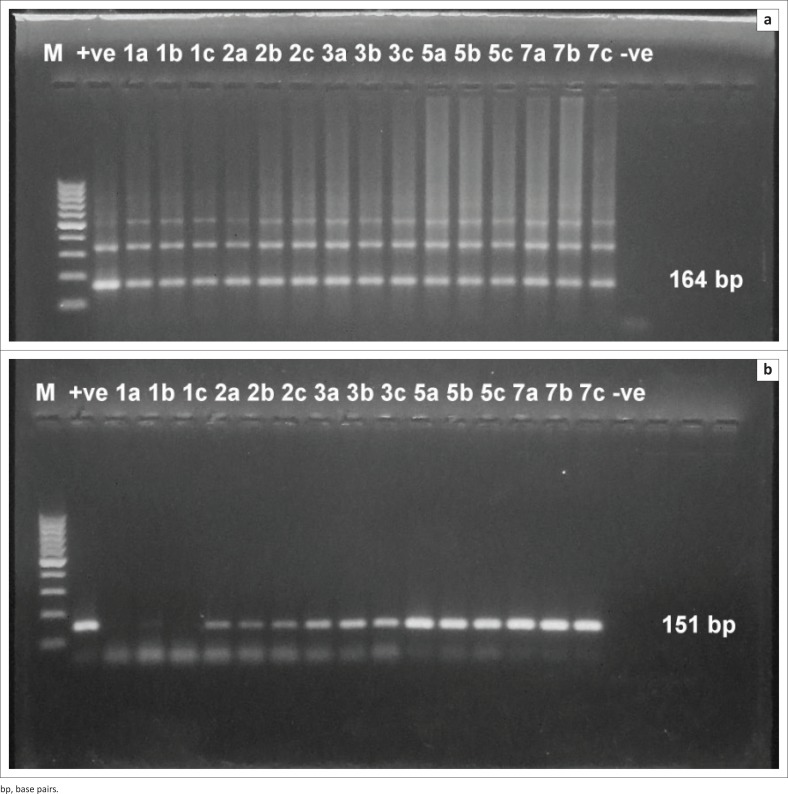
Direct polymerase chain reaction from mouse injected with 10^2^ trypanosome. (a) Polymerase chain reaction amplification using *Trypanosoma brucei* 1/2 primer sets. (b) Polymerase chain reaction amplification using *T. evansi Rode Trypanozoon antigen type* 1.2 primer sets. M: 100 base pair molecular weight Deoxyribonucleic acid marker, +ve: positive control, -ve: negative control, Numbers: days post infection. Letters: mice were sacrificed at day 1, 3, 5 and 7 post infection.

It was found that the assay was able to detect *T. evansi* DNA at 1 DPI in both experiments. High detection sensitivity was obtained by using TBB1/2 primer sets (Figures 3a and 4a) with convenient band intensity on the agarose gel throughout the course of infection. However, by using RoTat1.2 primer sets, the intensity of the amplified bands was clearly observed and increased gradually from the first DPI until the peak of parasitaemia in both experiments (Figures 3b and 4b). It was observed that there was no variation in the results between female and male mice.

### Detection of *Trypanosoma evansi* in different female and male organs in both experiments

**FIGURE 5:**
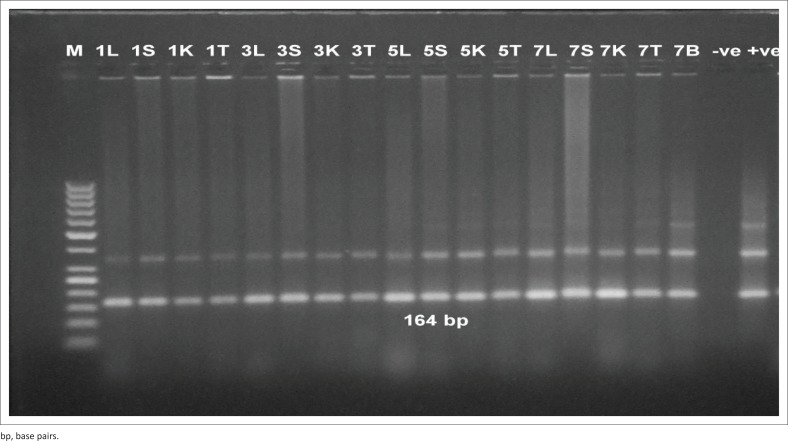
Polymerase chain reaction R amplification of Deoxyribonucleic acid extracted from tissues of infected male mice (parasitaemic stage) using *Trypanosoma brucei* primer set. M: 50 base pairs molecular weight Deoxyribonucleic acid marker, +ve: positive control, -ve: negative control, 1, 3, 5, 7: days post infection and L (liver, ) S (spleen), K (kidneys), T (testes) and B (brain).

**FIGURE 6:**
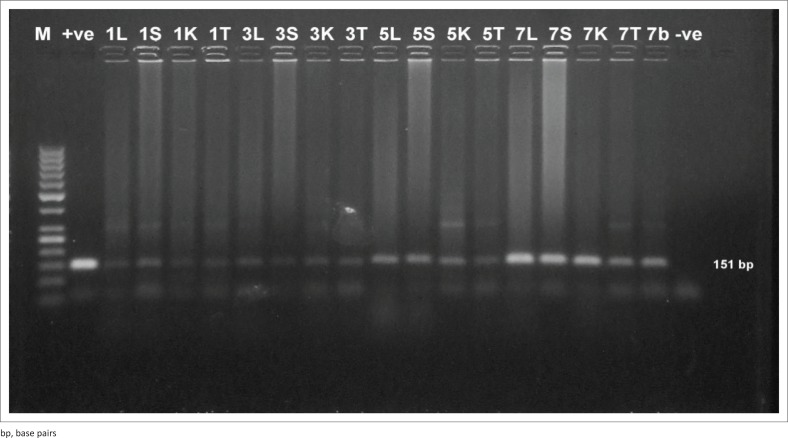
Polymerase chain reaction amplification of Deoxyribonucleic acid extracted from tissues of infected male mice (parasitaemic stage) using *T. evansi Rode Trypanozoon antigen type* primer set. M: 50 base pairs molecular weight Deoxyribonucleic acid marker, +ve: positive control, -ve: negative control, 1, 3, 5, 7: days post infection and L (liver), S (spleen), K (kidneys), T (testes) and B (brain).

**FIGURE 7:**
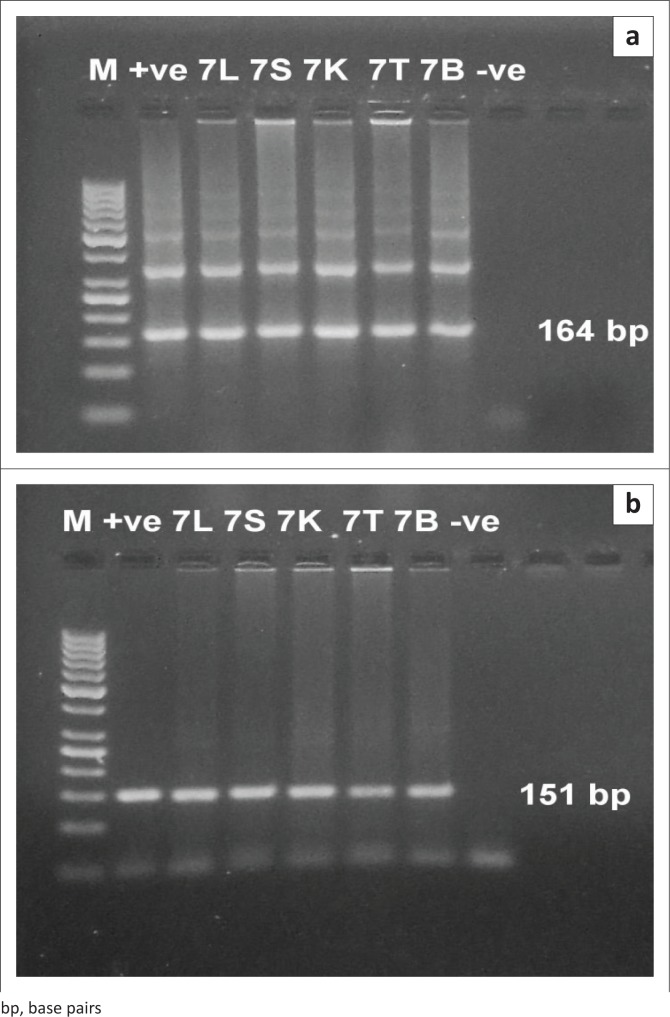
Polymerase chain reaction amplification of Deoxyribonucleic acid extracted from tissues of infected male mice (chronic) using (a) *Trypanosoma brucei* primer set and (b) *T. evansi* Rode Trypanozoon antigen type primer set. M: 50 base pairs molecular weight Deoxyribonucleic acid marker, +ve: positive control, -ve: negative control, 7: days post infection and L (liver), S (spleen), K (kidneys), T (testes) and B (brain).

The PCR technique could detect the infection at 24 hours post infection (PI) in both experiments in different examined organs, such as liver, spleen, kidney, testis and brain. The specific bands of the used primers appeared at 164 bp and 151 bp DNA fragments for TBR and TeRoTat primers, respectively. Furthermore, TBR1/2 primers displayed clear bands at 1 DPI, and TeRoTat1.2 primers showed a faint band at 1 DPI, with an increased band intensity as the infection progressed towards the parasitaemic stage (Figures 5 and 6). In addition, in the chronic stage, no variation was detected between the used primers (Figure 7).

### Detection and isolation of *Trypanosoma evansi* from clinical samples of camels

**FIGURE 8:**
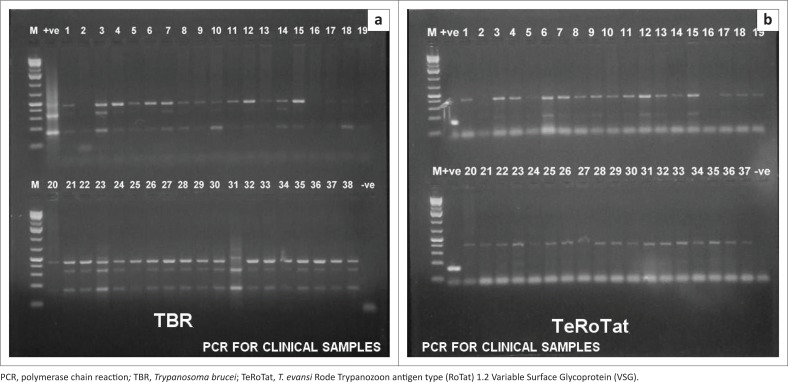
Polymerase chain reaction results of field blood samples with *Trypanosoma brucei* 1/2 (164 base pairs) and *T. evansi* Rode Trypanozoon antigen type (RoTat) 1.2. (151 base pairs) primers sets: M: 100 base pairs molecular weight Deoxyribonucleic acid marker, +ve: positive control *Trypanosoma evansi* Deoxyribonucleic acid and -ve: negative control. (a) TBR and (b) TeRoTat

All blood samples of camels were negative for *T. evansi* by MHCT or mouse inoculation. Meanwhile, PCR showed that all the tested samples were positive for the presence of the parasite using TBR1/2 primer sets. However, by using TeRoTat1.2 primer, only 32 out of 44 animals were positive for *T. evansi* infection (Figure 8).

## Discussion

Molecular diagnostic techniques represent essential tools for the detection of *T. evansi* infection and are widely spread in the detection of Surra globally because they are rapid, accurate and reliable (Desquesnes & Davila 2002; Sengupta et al. 2010). Monitoring parasitiaemic level in *T. evansi* is important for evaluating the health status of the host, determination of disease stage and risk of transmission of disease between animals in the field. Experimental infection of *T. evansi* was done in mice with two different inoculation doses to follow up the disease and determine which technique is more suitable technique for the early detection of *T. evansi*. Infection of mice with 10^4^ and 10^2^ trypanosomes in experiment I and experiment II, respectively, showed variable disease patterns, which could be ascribed to the inoculum dose and sex of mice. Microscopic examination by MHCT showed no difference in parasitaemia in the pre-patent periods between male and female mouse groups in experiment I 3 DPI. However, the progress of infection was delayed in male mice compared to female mice as the peaks of parasitaemia were achieved earlier in female than male mice. Differences in infection progress and parasitaemic peaks in *T. evansi* infection are sex dependant, and may be influenced by a stronger immune response against *T. brucei* in male mice compared to female ones Carvalho et al. (2018).

All remaining *Trypanosoma*-infected mice were died by the 9 DPI because of acute parasitaemia. In experiment II (10^2^ ID), the pre-patent period was delayed 2 days before compared to experiment I as a result of decreased inoculum dose. Similar results were observed by Sharma et al. (2012) after *T. evansi* experimental infection in mice, and the parasite was detected at 3 days and 4.5 days post-inoculation of 10^4^ and 10^2^ parasites, respectively.

The different patterns of infection observed after inoculation of 10^2^ parasites are a reflection of levels of immune response elicited among animals. Female mice showed consistent parasitaemia until death (G1), which can be ascribed to a depressed immune status usually observed in the acute form of the disease.

A persistent chronic undetectable infection in G2 is a reflection of adaptive immune resistance against infection, which represents a carrier state. Fluctuating parasitaemia throughout the course of infection in G3 mirrored antigenic variation among the strains of *T. evansi* and evasion of host immune response (Maudlin, Holmes & Miles 2004). The mechanism is a result of the parasite’s ability to periodically switch its major variant surface glycoprotein yielding a parasitaemic relapse (Herrera et al. 2004).

Two parasitaemic waves were observed in male mice. Most of males died from acute parasitaemia where only three mice showed one wave of parasitaemic disappearance and were dead by the second wave of parasitaemia. When the results of MHCT (parasitological test) and PCR (molecular technique) were compared, conventional PCR with TBR1/2 and TeRoTat1.2 primer sets was able to detect the parasite as early as 1 DPI in mice. However, MHCT detected parasite at 3 DPI in experiment I and at 5 DPI in experiment II. All tests were able to detect a peaking parasitaemia.

These results are in line with those of previous studies (Ashour et al. 2013; Sengupta et al. 2010). However, Fernandez et al. (2009) determined the pre-patency 12 hours post infection in mice by TBR1/2 and 1 DPI by ITS1 primers sets. Striking differences in the intensity of the bands amplified by TeRoTat1.2 primer sets from day 1 until the appearance of a consistent parasitaemia were ascribed to differences in the parasite count in the blood samples. González et al. (2006) and Fernandez et al. (2009) confirmed that the proportion of DNA is usually a reflection of the number of the parasite in a blood sample. Therefore, they recommended the use of at least 100 ng DNA in the sample when parasitaemia is lower than 10^3^ parasites/mL to ensure the detection of the parasite by PCR assay.

In TBR1/2 PCR, the intensity of the amplified bands showed little or no difference through the course of infection. This corresponded to the high number of repeat copies of the target sequence, which overcomes low parasitic DNA concentration in the sample.

In the current study, SYBR Green real-time PCR assay was carried out using TeRoTat1.2 primer sets, as described by Konnai et al. (2009). Conventional PCR results were able to detect the pre-patent periods (i.e. appearance of parasite in blood) at 1 DPI in both experiments I and II in mice. These results were further confirmed by melting curve analysis, which was rapid and avoided the downstream procedure of the conventional PCR. Taylor, Boyle and Bingham (2008) detected the parasite 6 DPI in rats injected with 10^4^ trypanosomes using ITS-1 TaqMan real-time PCR; however, Sharma et al. (2010) detected the parasite 1.5 DPI in mice infected with 10^4^ parasites and 3 DPI with 10^2^ parasites by the same assay. Discrepancies between our results and those obtained in previous studies may be because of variations in real-time conditions and different target sequences used in the techniques. Animals infected with *T. evansi* are characterised by fluctuating parasitaemia as a result of interactions between the host immune response and the ability of the parasite to evade immunity by antigenic variation. Parasitaemia is usually accompanied by the rise and fall of body temperature of the host (Dargantes et al. 2009). During these periods, the demonstration of *T. evansi* in the blood of animals is obvious; however, low-level parasitaemia results in the difficulty of parasite detection.

The polymerase chain reaction was able to detect *T. evansi* during the chronic phase of the disease in mice, while MHCT could not. The limited sensitivity and false-negative results of MHCT may be attributed to the fluctuation of parasitaemia and the presence of parasite with low number (Nantulya 1990). Our results are in agreement with those obtained by Ashour et al. (2013) and Ramírez-lglesias et al. (2011) in mice and rabbits, respectively.

Differences between our results and those obtained in previous studies may also be because of diversity of trypanosome strains, different PCR conditions, different primer sets and DNA extraction methods, as mentioned by Fernandez et al. (2009). In this work, Chelex resin was used for DNA extraction from blood samples. The method provided high DNA yield with convenient purity, and was less laborious and overcame the toxic effect and risk of using organic extraction methods (Herrera et al. 2005).

Application of PCR for *T. evansi* field diagnosis in camels showed variable results among the primers sets. The MHCT and mouse inoculation were not able to detect the parasite in the field samples. This might have been influenced by preservation methods and the duration transportation of samples before laboratory examination. In the same vein, Holland et al. (2001) reported that trypanosomes in samples from animals with high (>10^4^ trypanosomes/mL of blood) and low parasitaemia (250 trypanosomes/mL of blood) could not be detected beyond 8 and 3 h after storage at 4 °C and 27 °C, respectively. However, positive results were recorded for all samples using conventional PCR and TBR1/2 primer sets, where TeRoTat1.2 PCR was able to detect 32 out of 44 samples.

The low sensitivity of the RoTat1.2 PCR compared with that obtained by TBR1/2 was attributed to several factors such as number of copies of the target DNA sequence on the genome, parasitaemic level, parasitic DNA concentration and non-RoTat1.2 (Variable Surface Glycoprotein) VSG *T. evansi* variant existence that was previously reported in Kenya (Sengupta et al. 2010). Similar results were obtained by Elhaig et al. (2013), who reported that TBR primers showed higher sensitivity and specificity than five other primer sets for the detection of *T. evansi* and were able to detect parasitaemia below one parasite per millilitre of blood.

In conclusion, this study demonstrated that PCR was accurate, sensitive and reliable in the detection of the early *T. evansi*-infected mice, and also able to discriminate chronically infected carrier animals. In addition, PCR with TBR1/2 was more sensitive than TeRoTat1.2. However, TBR1/2 could detect field infection even when samples were negative by conventional methods. Therefore, this study will contribute towards understanding the course of the disease and finding suitable diagnostic tools for *T. evansi*. Furthermore, the use of molecular PCR for screening of newly introduced animals will help in excluding the carrier animals and detecting the early infected animals for saving free herds.
